# Chloride intracellular channel 1 as a switch among tumor behaviors in human esophageal squamous cell carcinoma

**DOI:** 10.18632/oncotarget.25296

**Published:** 2018-05-01

**Authors:** Toshiyuki Kobayashi, Atsushi Shiozaki, Yoshito Nako, Daisuke Ichikawa, Toshiyuki Kosuga, Katsutoshi Shoda, Tomohiro Arita, Hirotaka Konishi, Shuhei Komatsu, Takeshi Kubota, Hitoshi Fujiwara, Kazuma Okamoto, Mitsuo Kishimoto, Eiichi Konishi, Yoshinori Marunaka, Eigo Otsuji

**Affiliations:** ^1^ Division of Digestive Surgery, Department of Surgery, Kyoto Prefectural University of Medicine, Kyoto, 602-8566, Japan; ^2^ Department of Gastrointestinal, Breast & Endocrine Surgery, Faculty of Medicine, University of Yamanashi, Chuo, 409-3898, Japan; ^3^ Department of Pathology, Kyoto Prefectural University of Medicine, Kyoto, 602-8566, Japan; ^4^ Departments of Molecular Cell Physiology and Bio-Ionomics, Graduate School of Medical Science, Kyoto Prefectural University of Medicine, Kyoto, 602-8566, Japan; ^5^ Japan Institute for Food Education and Health, St. Agnes’ University, Kyoto, 602-8013, Japan

**Keywords:** CLIC1, esophageal squamous cell carcinoma, TLR2/JNK pathway, tumor survival, cellular movement

## Abstract

*Background:* Recent studies have reported important roles for chloride intracellular channel 1 (CLIC1) in various cancers; however, its involvement in esophageal squamous cell carcinoma (ESCC) remains unclear. The aim of the present study was to investigate the role of CLIC1 in human ESCC. *Methods*: CLIC1 expression in human ESCC cell lines was analyzed by Western blotting. Knockdown experiments were conducted with CLIC1 siRNA, and their effects on cell proliferation, the cell cycle, apoptosis, migration, and invasion were analyzed. The gene expression profiles of cells were analyzed using a microarray analysis. An immunohistochemical analysis was performed on 61 primary tumor samples obtained from ESCC patients who underwent esophagectomy. *Results:* ESCC cells strongly expressed CLIC1. The depletion of CLIC1 using siRNA inhibited cell proliferation, induced apoptosis, and promoted cell migration and invasion. The results of the microarray analysis revealed that the depletion of CLIC1 regulated apoptosis via the TLR2/JNK pathway. Immunohistochemistry showed that CLIC1 was present in the cytoplasm of carcinoma cells, and that the very strong or very weak expression of CLIC1 was an independent poor prognostic factor. *Conclusions:* The present results suggest that the very strong expression of CLIC1 enhances tumor survival, while its very weak expression promotes cellular movement. The present study provides an insight into the role of CLIC1 as a switch among tumor behaviors in ESCC.

## INTRODUCTION

Chloride intracellular channel 1 (CLIC1) is one of the CLIC family proteins, and was initially found to be overexpressed in activated macrophages [1]. CLIC1 exists as an integral membrane form and soluble cytoplasmic form [2]. As a homologous protein of the glutathione S-transferase (GST) superfamily [3, 4], CLIC1 is considered to act as a sensor and effector during oxidative stress [5]. CLIC1 is ubiquitously expressed in human tissues, and plays important roles in many physiological functions, including the modulation of ion homeostasis and regulation of cell volume and organelle acidity [6-8]. Recent studies revealed that CLIC1 is expressed in various cancers, and plays crucial roles in multiple cell functions including control of the cell cycle, apoptosis, proliferation, invasiveness, and metastasis [8-24]. However, the expression and role of CLIC1 in human esophageal squamous cell carcinoma (ESCC) remains unknown.

We previously reported that several ion transporters are expressed in human ESCC and play crucial roles in multiple cell functions. For example, Na^+^/K^+^/2Cl^-^ cotransporter 1 (NKCC1) regulates cell cycle progression [25], K-Cl cotransporter 3 (KCC3) regulates cell migration and invasion [26], anion exchanger 1 (AE1) functions as a regulator of proliferation, survival, migration, and invasion [27], Na^+^/H^+^ exchanger 1 (NHE1) plays a suppressive role in proliferation, survival, migration, and invasion [28], and aquaporin 5 (AQP5) affects proliferation and cell survival [29]. The objectives of the present study were to investigate the role of CLIC1 in the proliferation, apoptosis, migration, and invasion of ESCC. Microarray analyses showed that the depletion of CLIC1 with small interfering RNA (siRNA) changed the expression levels of many genes involved in apoptosis and epithelial-mesenchymal transition (EMT). Furthermore, we analyzed the expression of CLIC1 in human ESCC samples and investigated its relationship with the clinicopathological features and prognosis of ESCC patients. Our results indicate that CLIC1 plays an important role in the progression of ESCC.

## RESULTS

### Expression of CLIC1 in ESCC cell lines

**Figure 1:**
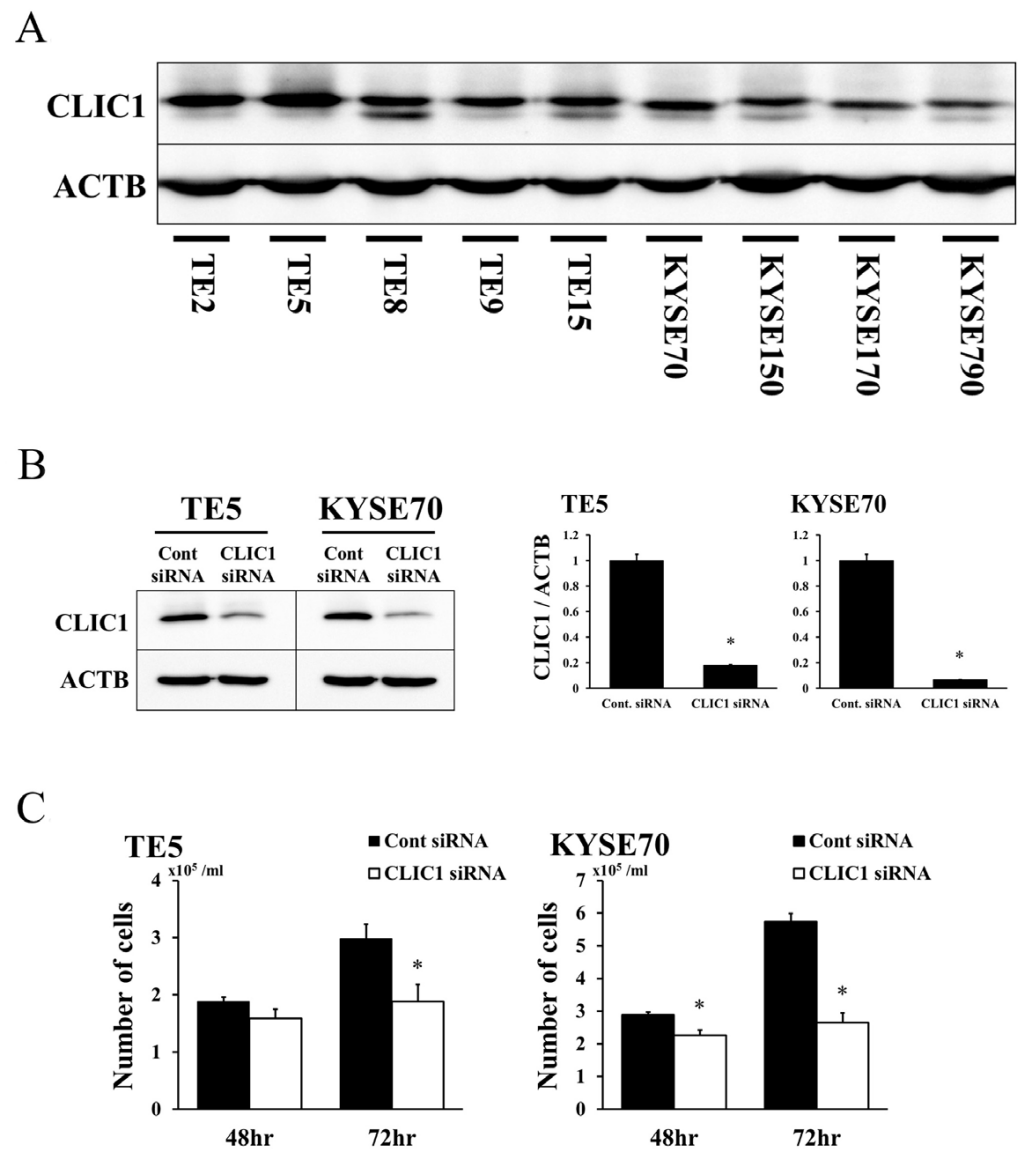
Expression of CLIC1 in ESCC cells **(A)** CLIC1 protein expression was analyzed in 9 ESCC cell lines. Western blotting showed that CLIC1 was strongly expressed in all 9 ESCC cell lines. **(B)** Western blotting revealed that CLIC1 siRNA effectively reduced CLIC1 protein levels in TE5 and KYSE70 cells. CLIC1 siRNA effectively reduced CLIC1 mRNA levels in TE5 and KYSE70 cells. Mean±SEM. n=3. ^*^p<0.05 (significantly different from control siRNA). **(C)** The down-regulation of CLIC1 inhibited the proliferation of TE5 and KYSE70 cells. The number of cells was counted 48 and 72 h after siRNA transfection. Mean±SEM. n=3. ^*^p<0.05 (significantly different from control siRNA).

In order to elucidate the functions of CLIC1 in ESCCs, we investigated 9 cell lines for CLIC1 protein expression. Western blotting revealed that the CLIC1 protein was expressed in all cell lines (Figure 1A).

### CLIC1 regulates cell proliferation in ESCC cells

**Figure 2:**
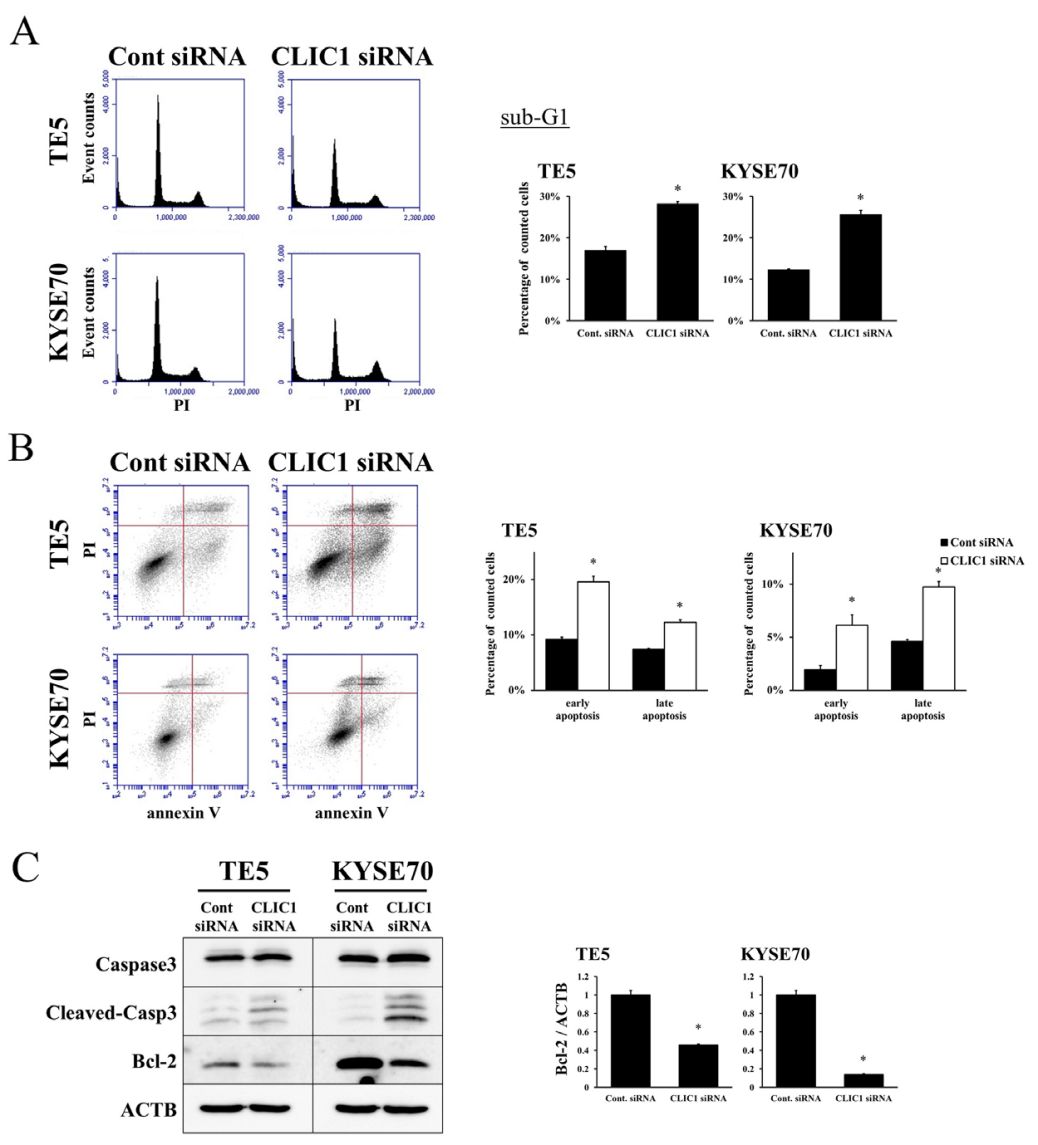
CLIC1 controls apoptosis of ESCC cells **(A)** The down-regulation of CLIC1 increased the number of cells in the sub-G1 phase in TE5 and KYSE70 cells. Cells transfected with control or CLIC1 siRNA were stained with propidium iodide (PI) and analyzed by flow cytometry. Mean±SEM. n=3. ^*^p<0.05 (significantly different from control siRNA). **(B)** The down-regulation of CLIC1 increased the early and late apoptosis phases in TE5 and KYSE70 cells. Cells transfected with control or CLIC1 siRNA were stained with PI and annexin V, and then analyzed by flow cytometry. Mean±SEM. n=3. ^*^p<0.05 (significantly different from control siRNA). **(C)** The down-regulation of CLIC1 affected reductions in Bcl-2 protein and mRNA levels and the cleavage of caspase 3.

We performed knockdown experiments using CLIC1 siRNA on the TE5 and KYSE70 cell lines and investigated their effects on cell proliferation. CLIC1 protein and mRNA levels were markedly decreased by CLIC1 siRNA transfection in both cell lines (Figure 1B). Cell numbers were significantly lower in CLIC1 siRNA-transfected TE5 cells than in control cells 72 h after transfection (Figure 1C). In KYSE70 cells, the number of CLIC1-depleted cells was significantly lower than that of control cells 48 and 72 h after transfection (Figure 1C). In the cell cycle analysis, the knockdown of CLIC1 increased the number of cells in the sub-G1 phase in the TE5 and KYSE70 cell lines (Figure 2A). These results indicate that CLIC1 has a critical function in controlling the proliferation and survival of ESCC cells.

### CLIC1 regulates apoptosis in ESCC cells

We then transfected TE5 and KYSE70 cells with CLIC1 siRNA and examined apoptosis. The depletion of CLIC1 increased early apoptosis (annexin V positive/PI negative) and late apoptosis (annexin V positive/PI positive) in the TE5 and KYSE70 cell lines 72 h after siRNA transfection (Figure 2B). Western blotting and quantitative RT-PCR revealed that the down-regulation of CLIC1 affected reductions in Bcl-2 protein and mRNA levels, and the cleavage of caspase 3 (Figure 2C). These results suggest that the expression of CLIC1 affects apoptosis in ESCC cells.

### Depletion of CLIC1 promotes cell migration and invasion in ESCC cells

**Figure 3:**
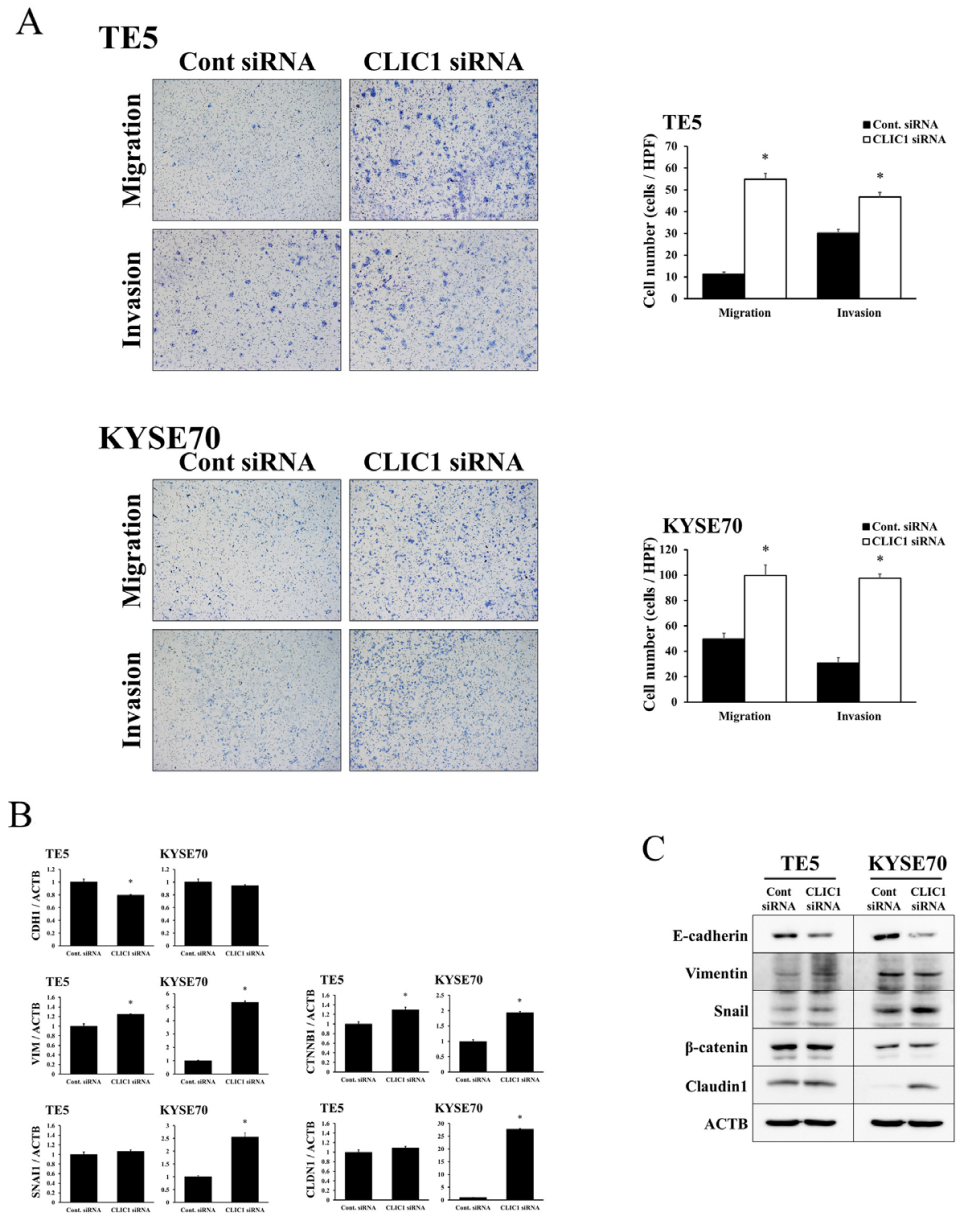
CLIC1 controlled the migration and invasion of ESCC cells **(A)** The down-regulation of CLIC1 significantly promoted the migration and invasion of TE5 and KYSE70 cells. Cell migration and invasion were assessed by the Boyden chamber assay. Magnification: ×40. Mean±SEM. n=3. ^*^p<0.05 (significantly different from control siRNA). **(B)** Verification of gene expression by real-time quantitative RT-PCR. The expression levels of five EMT-related genes (CDH1, VIM, SNAI1, CTNNB1, and CLDN1) in CLIC1-depleted TE5 and KYSE70 cells were compared to those in control siRNA-transfected cells using real-time quantitative RT-PCR. Gene expression levels were normalized to the level of ACTB. Mean±SEM. n=3. ^*^p<0.05 (significantly different from control siRNA). **(C)** Western blotting showed that the down-regulation of CLIC1 affected EMT-related factors. Detection of EMT-related proteins, including E-cadherin, vimentin, snail, β-catenin, and claudin 1, in CLIC1-knockdown TE5 and KYSE70 cells. The down-regulation of CLIC1 inhibited E-cadherin and induced snail and claudin 1.

The depletion of CLIC1 significantly increased the migration and invasion of TE5 and KYSE70 cells (Figure 3A). Western blotting and quantitative RT-PCR showed that the down-regulation of CLIC1 affected EMT-related molecular factors, such as E-cadherin, vimentin, snail, β-catenin, and claudin 1 (Figure 3B-3C). These results indicate that CLIC1 has critical functions in controlling cell migration and invasion in esophageal cancer. In terms of tumor behavior, the molecular mechanisms underlying cell migration and invasion may differ from those of apoptosis.

### Microarray analysis in CLIC1 siRNA-transfected ESCC cells

We examined the gene expression profiles of CLIC1 siRNA-transfected KYSE70 cells in the microarray analysis. The results obtained revealed that the expression of 3099 genes showed fold changes of > 3.0 in the KYSE70 cell line following the knockdown of CLIC1. Among these genes, 957 were up-regulated and 2142 were down-regulated in the CLIC1 siRNA-transfected KYSE70 cell line. Table 1 shows a list of the 20 genes with expression levels that were strongly up- or down-regulated in the CLIC1 siRNA-transfected KYSE70 cell line.

**Table 1:** Twenty genes displaying the greatest change in expression levels in CLIC1-depleted KYSE70 cells

Up-regulated Genes			
Gene Symbol	Gene ID	Gene Name	Fold Change
DAPP1	NM_014395	Dual adaptor of phosphotyrosine and 3-phosphoinositides	188.63
GUCY1B3	NM_000857	Guanylate cyclase 1, soluble, beta 3	148.50
C11orf88	NM_207430	Chromosome 11 open reading frame 88	145.68
LRCH1	NM_015116	Leucine-rich repeats and calponin homology (CH) domain containing 1	113.46
C3orf80	NM_001168214	Chromosome 3 open reading frame 80	107.61
EDA2R	NM_001242310	Ectodysplasin A2 receptor	85.48
MYD88	NM_002468	Myeloid differentiation primary response 88	80.89
ARG1	NM_001244438	Arginase 1	76.83
HSD11B1	NM_181755	Hydroxysteroid (11-beta) dehydrogenase 1	76.06
CPXM2	NM_198148	Carboxypeptidase X (M14 family), member 2	74.54
SLC40A1	NM_014585	Solute carrier family 40 (iron-regulated transporter), member 1	74.37
SIRPD	NM_178460	Signal-regulatory protein delta	74.11
NOXRED1	NM_001113475	NADP-dependent oxidoreductase domain containing 1	72.68
C21orf62	NM_019596	Chromosome 21 open reading frame 62	71.03
RHBG	NM_001256395	Rh family, B glycoprotein	70.75
LBP	NM_004139	Lipopolysaccharide binding protein	68.21
WFDC1	NM_021197	WAP four-disulfide core domain 1	67.19
IRF7	NM_004031	Interferon regulatory factor 7	59.49
PPARG	NM_138711	Peroxisome proliferator-activated receptor gamma	57.45
DPPA5	NM_001025290	Developmental pluripotency associated 5	56.87

Down-regulated Genes			
Gene Symbol	Gene ID	Gene Name	Fold Change
TRIM72	NM_001008274	Tripartite motif containing 72	-73.84
MYOT	NM_006790	Myotilin	-72.81
LDB3	NM_001171611	LIM domain binding 3	-71.29
OCLM	NM_022375	Oculomedin	-62.79
ESYT3	NM_031913	Extended synaptotagmin-like protein 3	-62.19
RPS4Y2	NM_001039567	Ribosomal protein S4, Y-linked 2	-58.48
COL5A2	NM_000393	Collagen, type V, alpha 2	-58.08
THEM4	NM_053055	Thioesterase superfamily member 4	-55.60
PPBP	NM_002704	Pro-platelet basic protein	-55.19

PGPEP1	NM_017712	Pyroglutamyl-peptidase I	-54.54
IKZF3	NM_012481	IKAROS family zinc finger 3	-51.05
CLCN4	NM_001830	Chloride channel, voltage-sensitive 4	-47.59
FAM92B	NM_198491	Family with sequence similarity 92, member B	-45.21
DYNLRB2	NM_130897	Dynein, light chain, roadblock-type 2	-44.66
SYT16	NM_031914	Synaptotagmin XVI	-43.77
CSF1R	NM_005211	Colony stimulating factor 1 receptor	-43.55
UNC5C	NM_003728	Unc-5 homolog C	-42.89
RXFP2	NM_130806	Relaxin/insulin-like family peptide receptor 2	-42.77
SULT6B1	NM_001032377	Sulfotransferase family, cytosolic, 6B, member 1	-42.23
ACRV1	NM_001612	Acrosomal vesicle protein 1	-42.05

IPA revealed that “Cancer” was one of the top-ranking diseases, and also that “Cell Morphology”, “Cellular Growth and Proliferation”, “Cellular Development”, “Cellular Movement”, and “Cellular Assembly and Organization” were top-ranking biological functions related to the depletion of CLIC1 (Supplementary Table 1).

### Signal pathways and molecular mechanisms regulated by CLIC1 in ESCC cells

**Figure 4:**
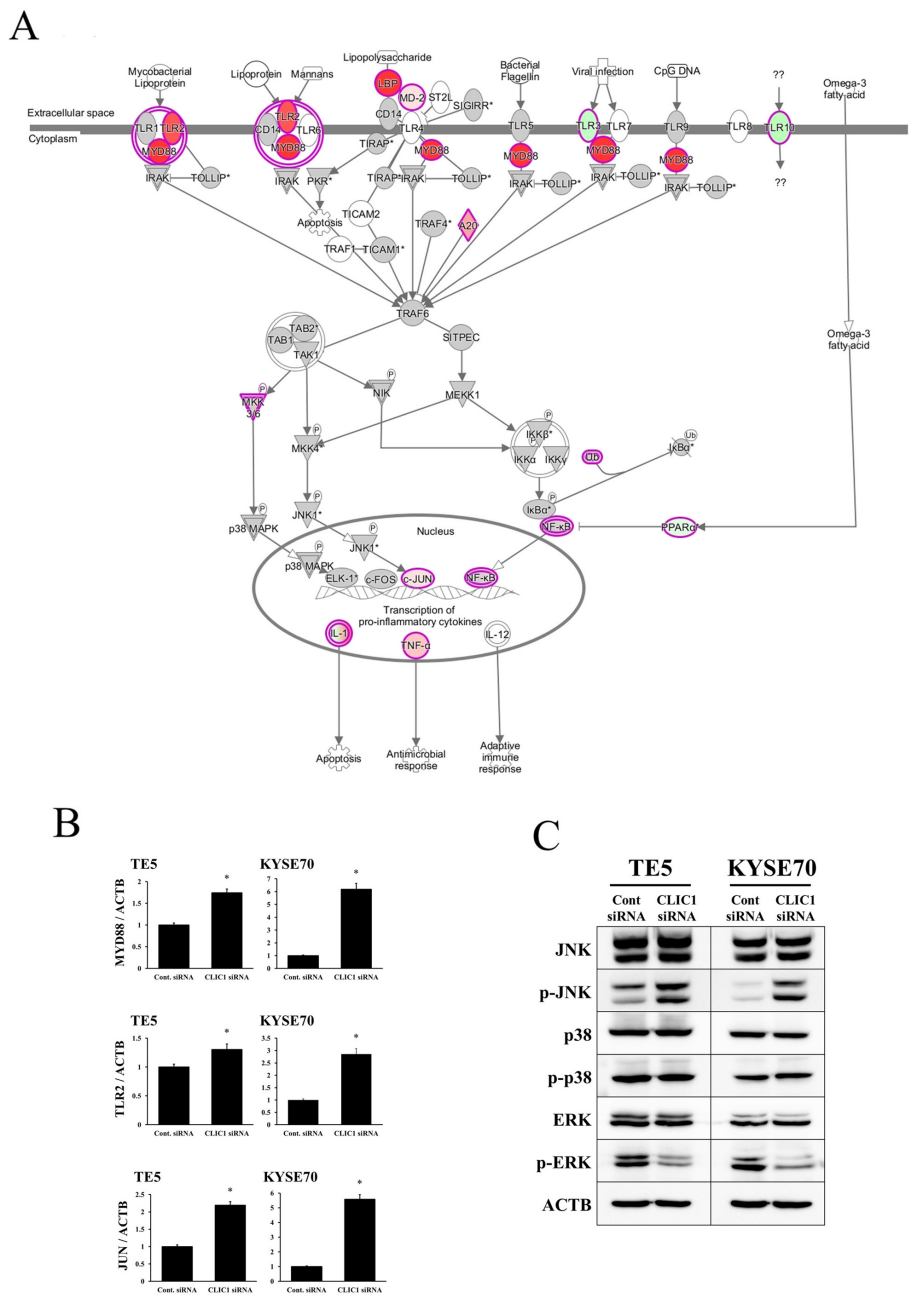
Top-ranked signaling pathway related to CLIC1 down-regulation according to an Ingenuity Pathway Analysis **(A)** The Toll-like receptor (TLR) signaling pathway was one of the top-ranked canonical pathways, and several genes related to this pathway were up-regulated in CLIC1-depleted KYSE70 cells. **(B)** Verification of gene expression by real-time quantitative RT-PCR. The expression levels of three selected genes (MYD88, TLR2, and JUN) in CLIC1-depleted TE5 and KYSE70 cells were compared to those in control siRNA-transfected cells using real-time quantitative RT-PCR. Gene expression levels were normalized to the level of ACTB. Mean±SEM. n=3. ^*^p<0.05 (significantly different from control siRNA). **(C)** Detection of the phosphorylation of JNK, p38, and ERK in CLIC1-knockdown TE5 and KYSE70 cells. Western blotting revealed that the down-regulation of CLIC1 activated the JNK pathway and inhibited the ERK pathway.

IPA showed that the “Toll-like receptor (TLR) signaling pathway” was one of the top-ranked canonical pathways, and also that several genes related to this pathway were up-regulated in CLIC1-depleted KYSE70 cells (Figure 4A, Table 2). In order to verify the data for gene expression profiling, 3 selected genes (MYD88, TLR2, and JUN) were examined in more detail using quantitative RT-PCR. The mRNA expression levels of MYD88, TLR2, and JUN were increased by CLIC1 siRNA transfection in TE5 and KYSE70 cells (Figure 4B). Figure 4A shows that the up-regulation of “JUN” required activation of the JNK pathway. Therefore, we focused on the TLR signaling pathway and MAPK pathway (not only “JNK”, but also “p38 MAPK” and “ERK”), and analyzed the functions of CLIC1 in the control of these pathways. A Western blot analysis revealed that the down-regulation of CLIC1 increased the phosphorylation level of JNK, decreased that of ERK, and did not affect that of p38 MAPK in TE5 and KYSE70 cells (Figure 4C). These results were consistent with those for gene expression profiles and indicated that the TLR signaling pathway and JNK pathway are key mechanisms by which CLIC1 controls cancer cell functions, such as the proliferation and survival of ESCC cells.

**Table 2:** Toll-like receptor signaling pathway-related genes with expression levels in KYSE70 cells that were changed by the depletion of CLIC1

Toll-like receptor signaling pathway			
Gene Symbol	Gene ID	Gene Name	Fold Change
MYD88	NM_002468	Myeloid differentiation primary response 88	80.89
LBP	NM_004139	Lipopolysaccharide binding protein	68.21
TLR2	NM_003264	Toll-like receptor 2	24.00
IL1B	NM_000576	Interleukin 1, beta	21.60
TNFAIP3	NM_006290	Tumor necrosis factor, alpha-induced protein 3	13.05
MAP2K6	NM_002758	Mitogen-activated protein kinase kinase 6	10.02
TNF	NM_000594	Tumor necrosis factor	8.25
UBD	NM_006398	Ubiquitin D	5.47
NFKB2	NM_001288724	Nuclear factor of kappa light polypeptide gene enhancer in B-cells 2	5.17
LY96	NM_015364	Lymphocyte antigen 96	4.25
IL1A	NM_000575	Interleukin 1, alpha	3.79
JUN	NM_002228	Jun proto-oncogene	3.10
IL33	NM_033439	Interleukin 33	-4.15
PPARA	NM_005036	Peroxisome proliferator-activated receptor alpha	-4.31
TLR3	NM_003265	Toll-like receptor 3	-7.79
TLR10	NM_030956	Toll-like receptor 10	-8.67

### Immunohistochemical analysis of CLIC1 expression in ESCC tumors

**Figure 5:**
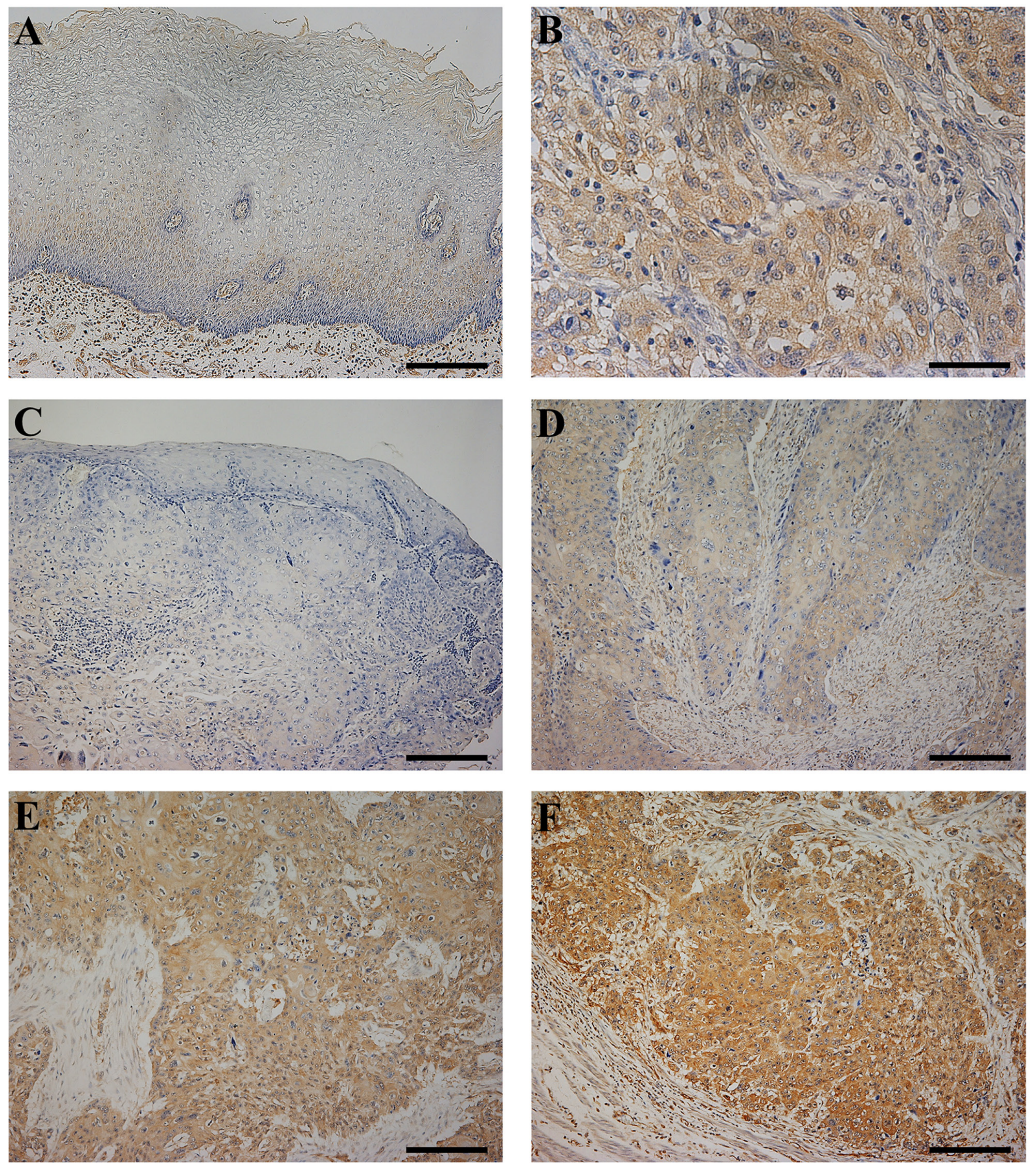
CLIC1 protein expression in human esophageal squamous cell carcinoma (ESCC) **(A)** Immunohistochemical staining of non-cancerous esophageal epithelia with the CLIC1 antibody. Magnification: ×100. Bar 200 μm. Cells expressing CLIC1 were primarily confined to the lower layer of the squamous epithelium with the exception of the basal and parabasal cell layers. CLIC1 expression of their lower layers was mainly weakly staining. **(B)** Immunohistochemical staining of primary human ESCC samples with the CLIC1 antibody. Magnification: ×400. Bar 50 μm. In ESCC cells, CLIC1 was expressed mainly in the cytoplasm. **(C-F)** Photomicrographs of CLIC1 immunohistochemistry are shown with examples of an intensity score of 0 (C), 1 (D), 2 (E), and 3 (F). Magnification: ×100. Bar 200 μm.

Immunohistochemistry for the CLIC1 protein revealed that CLIC1 expression was mainly observed in the cytoplasm of the lower layers of the non-cancerous esophageal epithelia, with the exception of the basal and parabasal cell layers (Figure 5A, Supplementary Figure 1A). In ESCC tissues, the CLIC1 protein was mainly expressed in the cytoplasm of cancer cells (Figure 5B). The CLIC1 expression levels of immunohistochemically stained samples were graded semi-quantitatively based on staining intensity and the percentage of positive tumor cells in the magnification ×100. Staining intensity was scored as either 0 (no staining), 1 (weakly staining), 2 (moderately staining), or 3 (strongly staining) (Figure 5C-5F, Supplementary Figure 1B-1E). Since in the normal esophageal epithelia, CLIC1 expression of their lower layers was mainly weakly staining, or the staining intensity score of 1 (Figure 5A, Supplementary Figure 1A), we focused on the region of the staining intensity score of 2 or 3 in ESCC tissues. We found the heterogeneity in the same ESCC tissue (Supplementary Figure 2). Therefore, the proportion of the staining intensity score of 2 or 3 was recorded as the “CLIC1 IHC score”. The median CLIC1 IHC score was 0.3 (range=0-1; mean±standard deviation (SD) = 0.42±0.36).

**Figure 6:**
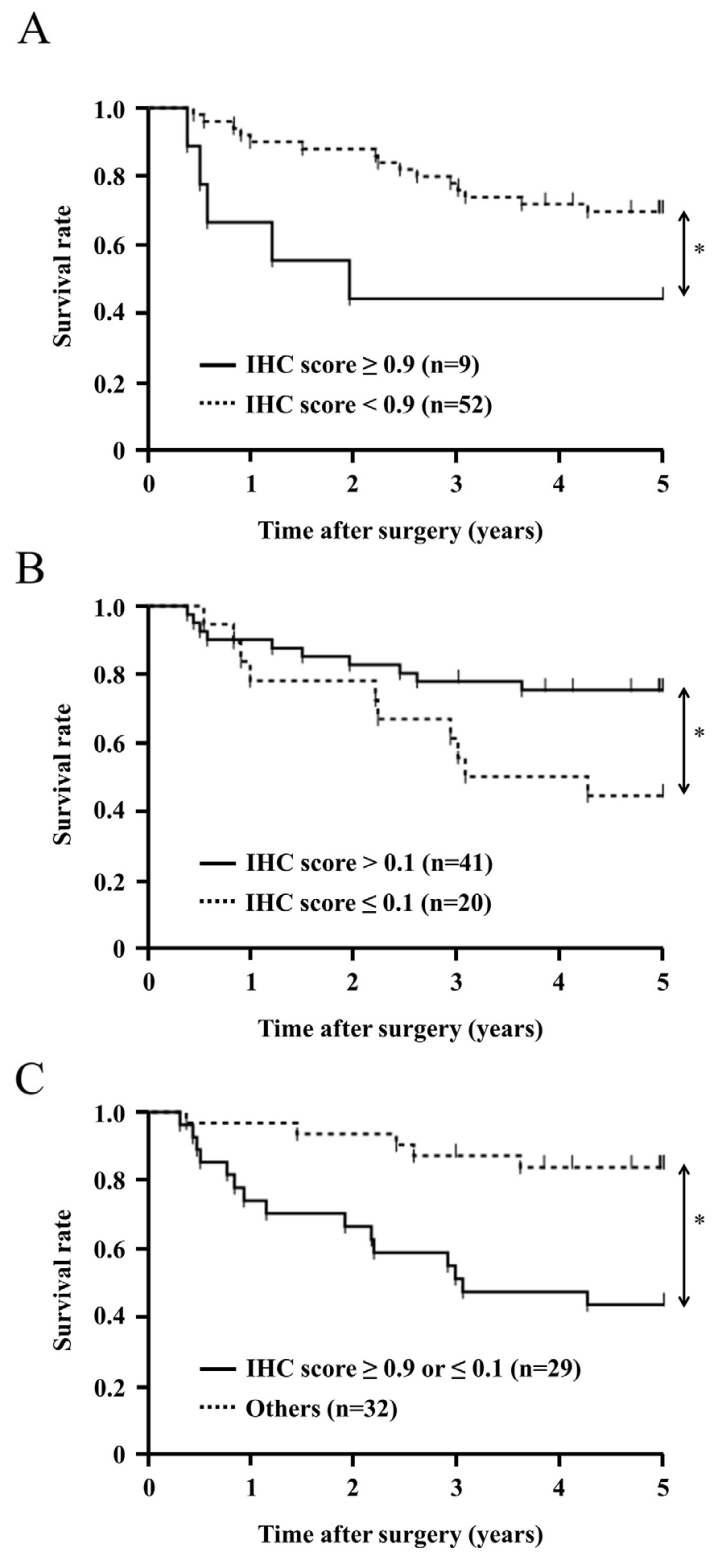
Survival curve of patients after curative resection for ESCC according to the expression of CLIC1 All patients were classified into two groups: **(A)** CLIC1 IHC score ≥0.9 (n=9), and CLIC1 IHC score <0.9 (n=52) in the tumor. **(B)** CLIC1 IHC score >0.1 (n=41), and CLIC1 IHC score ≤0.1 (n=20) in the tumor. **(C)** CLIC1 IHC score ≥0.9 or ≤0.1 (n=29), and CLIC1 IHC score <0.9 and >0.1 (n=32) in the tumor. ^*^p<0.05: Log-rank test.

Patients were initially categorized into a very strong CLIC1 expression group (IHC score ≥0.9, n=9) and the other (IHC score <0.9, n=52). In this analysis, no correlation was observed between clinicopathological features and the very strong expression of CLIC1 (Table 3). The 5-year overall survival rate of the very strong CLIC1 expression group was 44.4%, which was significantly poorer than that of the other group (69.8%) (p=0.041) (Figure 6B). These results support a relationship between the expression of CLIC1 and cell proliferation and apoptosis in *in vitro* experiments.

**Table 3:** Relationships between clinicopathological features of ESCC and CLIC1 expression

Variable	IHC score		p value ^*^	IHC score		p value ^*^
	< 0.9	≥ 0.9		≤ 0.1	> 0.1	
	(n=52)	(n=9)		(n=20)	(n=41)	
Gender						
Male	44	8	0.739	17	35	0.97
Female	8	1		3	6	
Age						
<65 years	32	3	0.114	15	20	0.048
≥65 years	20	6		5	21	
Recurrence						
Positive	19	5	0.281	10	14	0.236
Negative	33	4		10	27	
Tumor length						
<50 cm	36	5	0.42	13	28	0.798
≥50 cm	16	4		7	13	
Histological type						
Well/Moderately	37	7	0.682	10	34	0.008
Poorly	15	2		10	7	
Lymphatic invasion						
Negative	24	5	0.602	10	19	0.583
Positive	28	4		10	22	
Venous invasion						
Negative	26	6	0.355	11	21	0.781
Positive	26	3		9	20	
Pathological depth of the tumor						
pT1	22	5	0.46	8	19	0.639
pT2-3	30	4		12	22	
Pathological lymph node metastasis						
pN0	22	5	0.46	7	20	0.306
pN1-3	30	4		13	21	

^*^ Fisher’s exact test.

Patients were then categorized into a very weak CLIC1 expression group (IHC score **≤**0.1, n=20) and the other (IHC score >0.1, n=41). The very weak expression of CLIC1 correlated with age and histological type, but not with other clinicopathological variables, including gender, recurrence, tumor length, lymphatic invasion, venous invasion, pathological depth of the tumor, and pathological lymph node metastasis (Table 3). The 5-year overall survival rate of the very weak CLIC1 expression group was 42.4%, which was significantly poorer than that of the other group (77.4%) (p=0.034) (Figure 6A). These results support the depletion of CLIC1 promoting cellular movement in *in vitro* experiments.

Furthermore, Patients were categorized into three following groups; the very strong CLIC1 expression group (IHC score ≥0.9, n=9), the middle CLIC1 expression group (0.1< IHC score <0.9, n=32) and the very weak CLIC1 expression group (IHC score **≤**0.1, n=20). The 5-year overall survival rate of the very strong CLIC1 expression group and that of the very weak CLIC1 expression group were significantly poorer than that of the middle CLIC1 expression group (Supplementary Figure 3).

We investigated whether the very strong or very weak expression of CLIC1 was prognostic for ESCC patients after curative resection. The univariate analysis showed that the presence of lymphatic invasion, venous invasion, and the pathological depth of the tumor correlated with a poor 5-year overall survival rate. The 5-year overall survival rate of the very strong or very weak CLIC1 expression group was 44.8%, which was significantly poorer than that of the other group (84.2%) (p=0.001). A multivariate analysis with these three factors and an IHC score ≥0.9 or **≤**0.1 revealed that the very strong or very weak expression of CLIC1 was an independent prognostic factor (Table 4). These results suggest that very strong or very weak expression of CLIC1 in ESCC tissues is related to the poor prognosis of patients with ESCC after curative resection.

**Table 4:** Five-year overall survival rates of patients with ESCC according to various clinicopathological parameters

Variable		Univariate		Multivariate		
	n=61	5-year OS rate (%)	p value ^*^	HR	95% CI	p value ^**^
Gender						
Male	52	64.6	0.538			
Female	9	75				
Age						
<65 years	26	76	0.182			
≥65 years	35	58.8				
Tumor length						
<50 cm	41	67.1	0.591			
≥50 cm	20	64.3				
Histological type						
Well/Moderately	44	71.6	0.192			
Poorly	17	52.9				
Lymphatic invasion						
Positive	32	57.9	0.099	2.51	0.921-7.374	0.0724
Negative	29	74.8				
Venous invasion						
Positive	32	52.5	0.045	3.203	1.206-9.175	0.0192
Negative	29	77.6				
Pathological depth of the tumor						
pT1	27	77.4	0.054	2.694	0.964-8.299	0.0589
pT2-3	34	56.6				
Pathological lymph node metastasis						
pN0	27	73.5	0.207			
pN1-3	34	59.7				
IHC score						
≤ 0.1 or ≥ 0.9	29	44.8	0.001	10.31	3.542-35.70	<0.0001
Others	32	84.2				

OS: overall survival; HR: hazard ratio; CI: confidence interval.

^*^ Log-rank test. ^**^ Cox’s proportional hazards model.

## DISCUSSION

Recent studies have shown that CLIC1 is present in various tumor tissues, and demonstrated the expression and role of CLIC1 during tumor development in various cancers, including gastric cancer [9-11], colonic cancer [8, 12], hepatic cancer [13-15], gallbladder cancer [16, 17], pancreatic cancer [18], lung cancer [19], prostate cancer [20], ovarian cancer [21, 22], and glioblastoma [23]. However, the expression of CLIC1 in human ESCC tissues and the pathophysiological role of its expression in ESCC cells currently remain unclear.

The expression of CLIC1 correlates with cell proliferation and apoptosis in several cancers. Ma PF et al. [10] showed that the strong expression of CLIC1 inhibited cell proliferation and enhanced apoptosis in gastric cancer cells. Lu J et al. [18] reported that the depletion of CLIC1 inhibited cell proliferation and enhanced apoptosis in pancreatic cancer cells. In the present study, we demonstrated that the depletion of CLIC1 inhibited cell proliferation and enhanced apoptosis in ESCC cells. A cell cycle analysis revealed that the down-regulation of CLIC1 increased the number of cells in the sub-G1 phase in ESCC cells. These results indicate that the expression of CLIC1 influences apoptosis in ESCC cells.

In order to investigate the molecular mechanisms underlying the roles of CLIC1 expression in tumor progression and apoptosis, microarrays and a pathway analysis using IPA were performed using CLIC1 siRNA-transfected KYSE70 cells. The results obtained revealed that the TLR signaling pathway was one of the top-ranked canonical pathways, and also that “MYD88” and “TLR2” were strongly up-regulated genes. Previous studies reported that the TLR signaling pathway correlated with apoptosis. Krysko DV et al. [30] identified TLR2 and TLR9 as sensors of apoptosis. Zou et al. [31] showed that the up-regulation of TLR2/MYD88 induced the activation of c-Jun via the mitogen-activated protein kinase (MAPK)/JNK pathway, and then induced apoptosis. Zhao et al. [32] demonstrated that activating the MAPK/JNK pathway induced autophagy and apoptotic cell death in colonic cancer cells. The present results revealed that the depletion of CLIC1 activated the TLR2/MYD88 pathway and MAPK/JNK pathway, and induced apoptosis in TE5 and KYSE70 ESCC cells. This is the first study to show that the expression of CLIC1 may influence the activation of the TLR2/JNK pathway in ESCC cells.

Previous studies reported that the expression of CLIC1 had a crucial influence on ERK and p38, which are the other main subgroups of MAPKs. Wang P et al. [12] and Tian Y et al. [20] showed that the down-regulation of CLIC1 suppressed cell migration and invasion via the MAPK/ERK pathway in colonic and prostate cancer cells. Zhao W et al. [11] found that the overexpression of CLIC1 induced cell migration and invasion via the p38 MAPK pathway in gastric cancer cells. The present results revealed that the depletion of CLIC1 promoted cell migration and invasion, suppressed the MAPK/ERK pathway, and had no influence on the p38 MAPK pathway in ESCC cells. Therefore, the molecular mechanisms underlying cell migration and invasion in CLIC1-depleted ESCC cells appear to differ from those in other CLIC1-regulated cancer cells. The main cause of the difference among ESCC and other cancers remains unknown, and further investigations on the underlying molecular mechanisms are needed.

Previous immunohistochemical studies showed that the expression of CLIC1 in human tumor tissue samples was associated with the clinicopathological features and prognosis of patients. Chen CD et al. [9] examined 56 samples from gastric cancer patients and showed that metastasis correlated with CLIC1 expression, and also that the strong expression group had a significantly poorer prognosis than the weak expression group. Zhang S et al. [13] examined 85 samples from hepatic tumor patients and showed that the strong expression of CLIC1 correlated with tumor size, distant metastasis, and a poor survival. Ding Q et al. [17] examined 75 samples from gallbladder cancer patients and found that the overexpression of CLIC1 was associated with the histological type, perineural invasion, and poor prognosis. Lu J et al. [18] examined 75 samples from pancreatic cancer patients and demonstrated that the strong expression of CLIC1 was associated with the histological type, tumor size, and poor prognosis. Our immunohistochemical study revealed no relationship between the very strong expression of CLIC1 and clinicopathological features; however, the 5-year overall survival rate was significantly poorer in the very strong CLICI expression group than in the other group and the very strong expression of CLIC1 was an independent prognostic factor in ESCC tissues. On the other hand, the immunohistochemical investigation also revealed that the very weak expression of CLIC1 was associated with lymphatic metastasis, the 5-year overall survival rate was significantly poorer in the very weak CLICI expression group than in the other group, and the very weak expression of CLIC1 was an independent prognostic factor in human ESCC tissues. In *in vitro* experiments with ESCC cells, the expression of CLIC1 regulated tumor behaviors, including cell proliferation, apoptosis, and cellular movement, and our immunohistochemical results supported those obtained in *in vitro* experiments; that is, the group of very strong CLIC1 expression was poorer prognosis due to inhibiting apoptosis of ESCC cells, and the group of very weak CLIC1 expression was poorer prognosis due to promoting cell movement of ESCC cells. In short, our results indicate that CLIC1 expression levels are related to the switching of the tumor behaviors of ESCC. Although a deeper understanding of CLIC1 expression and its heterogeneity in biopsy specimen is needed, further analyses may be helpful in the clinical use of CLIC1 IHC score as a preoperative biomarker in future.

In summary, we herein demonstrated that CLIC1 plays a role in the proliferation, apoptosis, and cellular movement of ESCC cells. Our microarray data also showed that CLIC1 affects the expression of other genes with functions related to cell proliferation and apoptosis. Immunohistochemistry revealed that the very strong or weak expression of CLIC1 in human ESCC tissue was related to the prognosis of ESCC patients. Although further investigations on the underlying molecular mechanisms are needed, the present results suggest that CLIC1 is a useful biomarker of tumor progression and/or a novel therapeutic target for the future treatment of ESCC.

## MATERIALS AND METHODS

### Cell lines, antibodies, and other reagents

The poorly differentiated human ESCC cell lines, TE2, TE5, and TE9, moderately differentiated human ESCC cell line, TE8, and well-differentiated human ESCC cell line, TE15, were obtained from the Cell Resource Center of Biomedical Research Institute of Development, Aging, and Cancer (Tohoku University, Sendai, Japan). The poorly differentiated human ESCC cell lines, KYSE70 and KYSE150, moderately differentiated human ESCC cell line, KYSE170, and well-differentiated human ESCC cell line, KYSE790, were obtained from Kyoto University (Kyoto, Japan). These cells were grown in RPMI-1640 medium (Nacalai Tesque, Kyoto, Japan) supplemented with 100 U/mL of penicillin, 100 μg/mL of streptomycin, and 10% fetal bovine serum (FBS). Cells were cultured in flasks and dishes in a humidified incubator at 37°C under 5% CO_2_ in air.

The following antibodies were used in the present study: a mouse monoclonal CLIC1 antibody (Abcam, Cambridge, MA, UK), rabbit monoclonal c-Jun N-terminal kinase (JNK), phosphorylated JNK, extracellular signal-regulated kinase (ERK), phosphorylated ERK, p38, phosphorylated p38, snail, β-catenin, caspase 3, cleaved caspase 3, and Bcl-2 antibodies, mouse monoclonal E-cadherin and vimentin antibodies, horseradish peroxidase (HRP)-conjugated mouse and anti-rabbit secondary antibodies (Cell Signaling Technology, Beverly, MA, UK), a claudin 1 antibody (Zymed Laboratories, San Francisco, CA, USA), and monoclonal Anti-β-actin (mouse IgG1 isotype) antibody (Sigma-Aldrich, St. Louis, MO, USA).

### siRNA transfection

Cells were transfected with 12 nmol/L CLIC1 siRNA (Stealth RNAi siRNA #HSS101987, Invitrogen, Carlsbad, CA, USA) using the Lipofectamine RNAiMAX reagent (Invitrogen), according to the manufacturer’s instructions. Medium containing siRNA was replaced with fresh medium after 24 h. Control siRNA (Stealth RNAi siRNA Negative Control; Invitrogen) was used as a negative control.

### Protein studies

Cells were lysed with M-PER lysis buffer, sonicated, and then centrifuged at 15,000 rpm for 10 min at 4°C to obtain supernatants (cell lysates). Protein concentrations were measured using the Protein Assay Rapid Kit (Wako Pure Chemical Industries, Osaka, Japan). After SDS-PAGE, proteins were transferred onto polyvinylidene difluoride (PVDF) membranes. The membranes were then incubated with appropriate primary antibodies at 4°C overnight. The antibody complexes were visualized by an enhanced chemiluminescence detection system (ImageQuant LAS 4000 mini; GE Healthcare, Buckinghamshire, UK), as directed by the manufacturer. A densitometric analysis was performed using ImageQuant TL (GE Healthcare), and the band intensity of each protein was normalized by that of β-actin for each cell line.

### Real-time quantitative RT-PCR

Total RNA was extracted using an RNeasy kit (Qiagen, Valencia, CA, USA). Messenger RNA (mRNA) expression was measured by quantitative real-time PCR (7300 Real-Time PCR System; Applied Biosystems, Foster City, CA, USA) using TaqMan Gene Expression Assays (Applied Biosystems) according to the manufacturer’s instructions.

Expression levels were measured for the following genes: CLIC1 (Hs00559461_m1), MYD88 (Hs01573837_g1), TLR2 (Hs02621280_s1), JUN (Hs01103582_s1), Bcl2 (Hs00608023_m1), CDH1 (Hs00170423_m1), VIM (Hs00185584_m1), SNAI1 (Hs00195591_m1), CTNNB1 (Hs00355049_m1), and CLDN1 (Hs00221623_m1) (Applied Biosystems). The expression of each gene was normalized against the housekeeping gene β-actin (ACTB, Hs01060665_g1; Applied Biosystems). Each assay was performed in triplicate.

### Cell proliferation

Cells were seeded on six-well plates at a density of 0.5×10^5^ cells per well and incubated at 37°C with 5% CO_2_. siRNA transfection was performed 24 h after cell seeding. Cells were detached from the flasks 48 and 72 h after siRNA transfection using 0.5 ml trypsin–EDTA solution, diluted in 1.5 ml RPMI medium, and then counted with a hemocytometer.

### Cell cycle analysis

The cell cycle phase was evaluated 72 h after siRNA transfection by fluorescence-activated cell scoring (FACS). Briefly, cells were treated with Triton X-100 and RNase, and nuclei were stained with propidium iodide (PI) prior to the measurement of DNA content using BD Accuri C6 FACS (BD Biosciences, Franklin Lakes, NJ). At least 30,000 cells were analyzed.

### Analysis of apoptotic cells

Cells were harvested 72 h after siRNA transfection and stained with fluorescein isothiocyanate-conjugated annexin V and phosphatidylinositol using the annexin V kit (Beckman Coulter, Brea, CA, USA) according to the manufacturer’s protocol. The proportion of apoptotic cells was analyzed by flow cytometry with BD Accuri C6 (BD Biosciences).

### Patients and primary tissue samples

Histologically proven primary ESCC tumor samples were obtained from 61 consecutive patients who underwent esophagectomy (potentially curative R0 resection) at Kyoto Prefectural University of Medicine (Kyoto, Japan) between 1998 and 2009. Tumor samples were embedded in paraffin after formalin fixation for 12 h. Patient eligibility criteria were as follows: (1) the presence of ESCC, (2) the absence of synchronous or metachronous cancers, and (3) a lack of preoperative radiation therapy. All patients gave written informed consent. Relevant clinicopathological and survival data were obtained from hospital records. Cancer recurrence was noted in 24 patients (39.3%) during the follow-up period. The initial recurrence pattern detected by imaging studies was classified as locoregional, distant, or both sites. Nineteen patients (31.2%) died of cancer recurrence, and 1 (1.6%) of another disease. The median follow-up period of all patients was 62.0 months (range, 4–157 months). Staging was primarily based on the International Union Against Cancer (UICC)/TNM Classification of Malignant Tumors (7^th^ edition).

### Immunohistochemistry

Paraffin sections (thickness of 4 μm) of tumor tissues were subjected to immunohistochemical staining for the CLIC1 protein using the avidin-biotin-peroxidase complex method. Briefly, paraffin sections were dewaxed in xylene and hydrated through a graded series of alcohols. Antigen retrieval was not performed. Endogenous peroxidases were quenched by incubating the sections for 30 min in 0.3 % H_2_O_2_. Sections were then treated with a protein blocker and incubated at 4°C overnight with the anti-CLIC1 (1:3000) antibody diluted in PBS. The avidin-biotin-peroxidase complex system (Vectastain ABC Elite kit; Vector Laboratories, Burlingame, CA, USA) was used with diaminobenzidine tetrahydrochloride for color development. Sections were counterstained with hematoxylin, dehydrated through a graded series of alcohols, cleared in xylene, and then mounted.

CLIC1 expression levels of immunohistochemically stained samples were graded semi-quantitatively, considering both the staining intensity and percentage of positive tumor cells. Staining intensity was scored as either 0 (no staining), 1 (weakly staining), 2 (moderately staining), or 3 (strongly staining). The proportion of the staining intensity score of 2 or 3 was recorded as the “CLIC1 IHC score”.

### Microarray sample preparation and hybridization

KYSE70 cells were transfected with control siRNA and CLIC1 siRNA (n = 1). Total RNA was extracted 48 h after siRNA transfection using an RNeasy kit (Qiagen). RNA quality was monitored with an Agilent 2100 Bioanalyzer (Agilent Technologies, Santa Clara, CA, USA). Cyanine-3 (Cy3)-labeled cRNA was prepared from 0.1 μg of total RNA using a Low Input Quick Amp Labeling Kit (Agilent) according to the manufacturer’s instructions. Samples were purified using RNeasy columns (Qiagen). A total of 0.60 μg of Cy3-labeled cRNA was fragmented and hybridized to an Agilent SurePrint G3 Human Gene Expression 8 × 60 K ver 2.0 Microarray for 17 h. Slides were washed and scanned immediately on an Agilent DNA Microarray Scanner (G2565CA) using the one-color scan setting for 8 × 60 K array slides.

### Processing of microarray data

Scanned images were analyzed with Feature Extraction Software 10.10 (Agilent) using default parameters to obtain background-subtracted and spatially detrended Processed Signal intensities. Signal transduction networks were analyzed using Ingenuity Pathway Analysis (IPA) software (Ingenuity Systems, Inc., Redwood City, CA, USA).

### Statistical analysis

Chi-squared or Fisher’s exact tests were used to evaluate differences between proportions, and the Student’s *t*-test was used to evaluate continuous variables. Survival curves were constructed by the Kaplan–Meier method, and differences in survival were examined using the Log-rank test. Cox’s proportional hazard model was used to identify prognostic factors. Differences were considered significant when the relevant p value was <0.05. These analyses were performed using the JMP statistical software package (JMP, version 12, SAS Institute Inc., Cary, NC, USA).

## SUPPLEMENTARY MATERIALS FIGURES AND TABLE


