# Supplementary material for: Chloride intracellular channel 1 as a switch among tumor behaviors in human esophageal squamous cell carcinoma
Source: Oncotarget. 2018 May 1;9(33):23237–52. doi: 10.18632/oncotarget.25296 (PMC5955400; doi:10.18632/oncotarget.25296)
Supplement: Supplementary file 1 [file oncotarget-09-23237-s001.pdf]

Chloride intracellular channel 1 as a switch among tumor behaviors in human esophageal squamous cell carcinoma

SUPPLEMENTARY MATERIALS

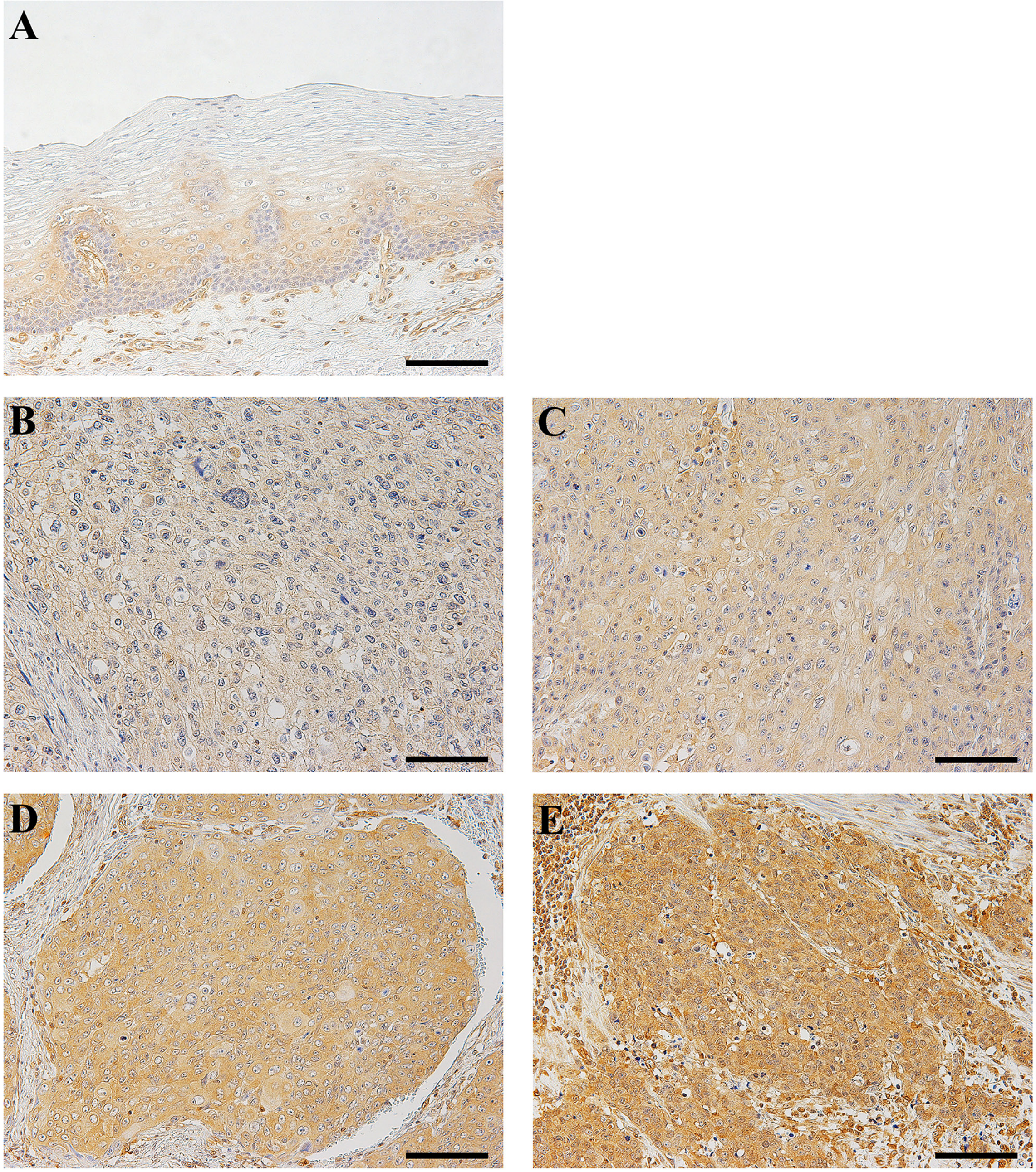

Supplementary Figure 1: CLIC1 protein expression in human esophageal squamous cell carcinoma (ESCC) using the high-power field. (A) Immunohistochemical staining of non-cancerous esophageal epithelia with the CLIC1 antibody. Magnification: ×200. Bar 100 µm. **(B-E)** Photomicrographs of CLIC1 immunohistochemistry are shown with examples of an intensity score of 0 (B), 1 (C), 2 (D), and 3 (E). Magnification: ×200. Bar 100 µm. In ESCC cells, CLIC1 was mainly expressed in the cytoplasm, but was not specifically expressed in cell membrane.

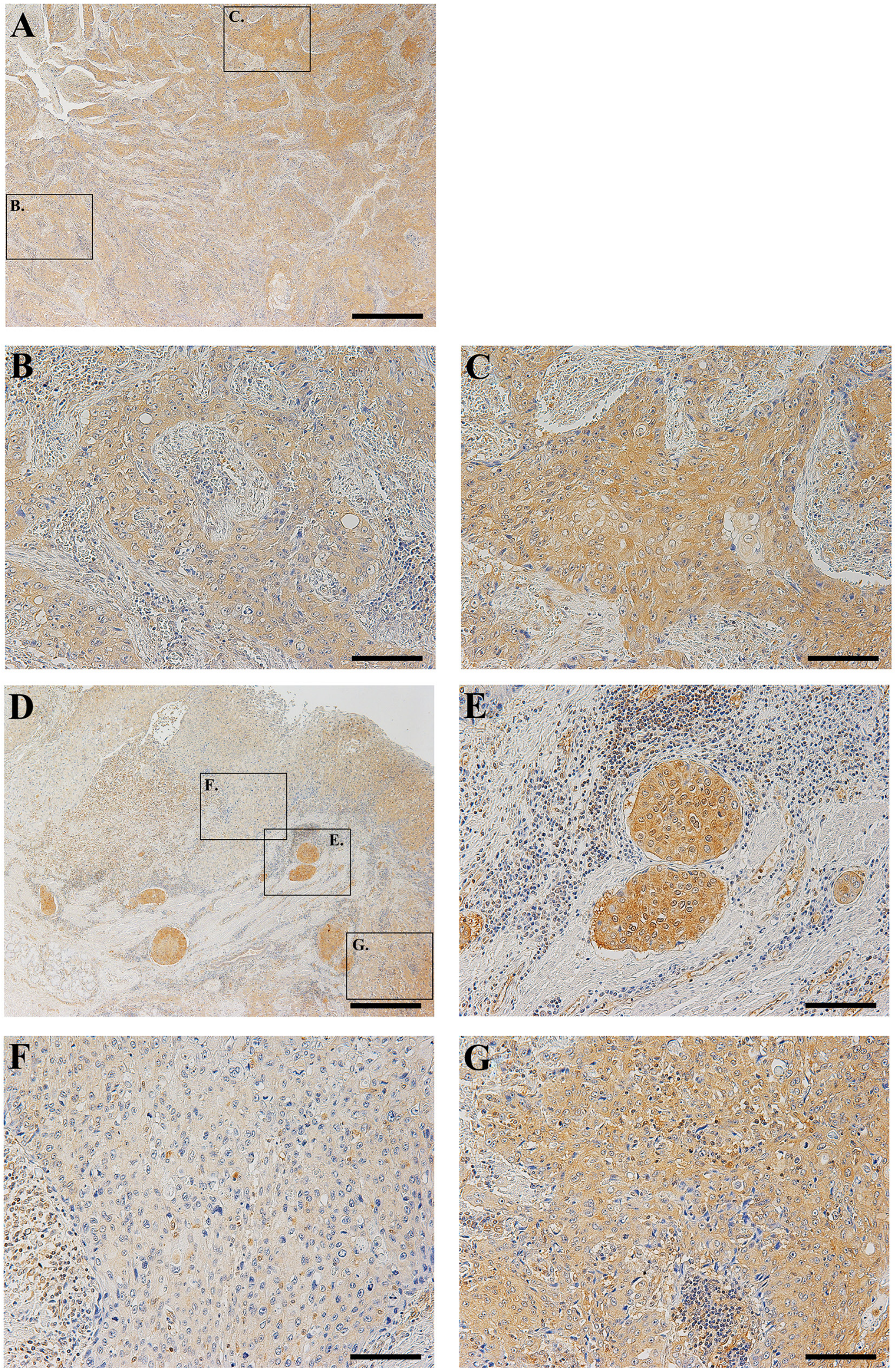

Supplementary Figure 2: Immunohistochemistry for CLIC1 showed that there was heterogeneity in resected ESCC tissues. **(A)** Immunohistochemical staining of one ESCC sample with the CLIC1 antibody. Magnification: ×40. Bar 500 μm. **(B)** The figure using the high-power field (magnification: ×200). Bar 100 μm. This area was weakly staining. **(C)** The figure using the high-power field (magnification: ×200). Bar 100 μm. This area was moderately staining. **(D)** Immunohistochemical staining of another ESCC sample with the CLIC1 antibody. Magnification: ×40. Bar 500 μm. **(E)** The figure using the high-power field (magnification: ×200). Bar 100 μm. This area was strongly staining. **(F)** The figure using the high-power field (magnification: ×200). Bar 100 μm. This area was no staining. **(G)** The figure using the high-power field (magnification: ×200). Bar 100 μm. This area was moderately staining.

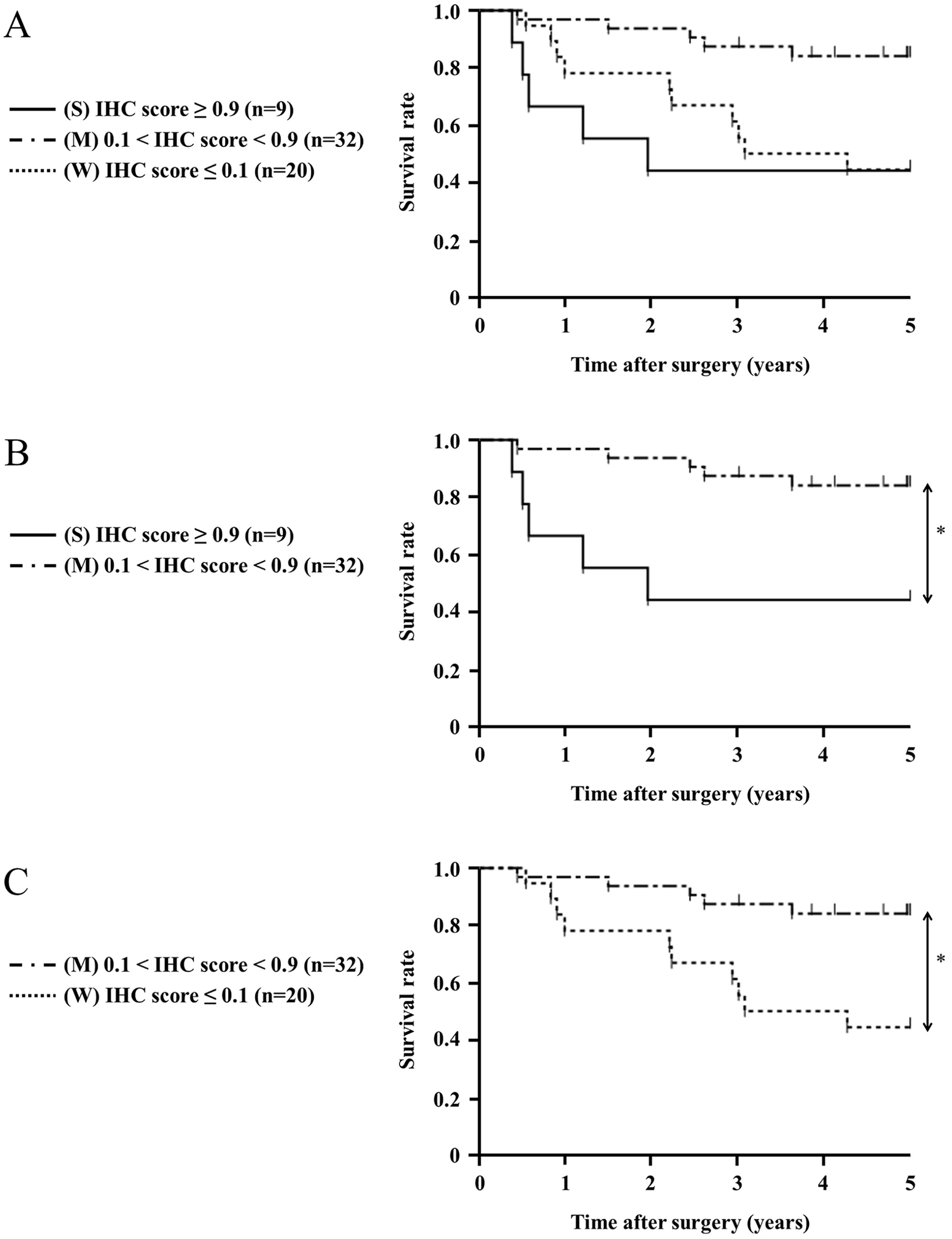

Supplementary Figure 3: Survival curve of patients after curative resection for ESCC among the three classified groups according to the expression of CLIC1. All patients were classified into three groups: (S) the very strong CLIC1 expression group, CLIC1 IHC score ≥0.9 (n=9); (M) the middle CLIC1 expression group, 0.1< IHC score <0.9 (n=32); (W) the very weak CLIC1 expression group, CLIC1 IHC score ≤0.1 (n=20) in the tumor. **(A)** Survival curve of patients after curative resection for ESCC among the three groups. **(B)** Survival curve of patients after curative resection for ESCC between group (S) and group (M). Group (S) was significantly poorer prognosis than group (M). **(C)** Survival curve of patients after curative resection for ESCC between group (M) and group (W). Group (W) was significantly poorer prognosis than group (M). $^{*}p<0.01$: Log-rank test.

Supplementary Table 1: Top diseases and biological functions of CLIC1 according to an Ingenuity Pathway Analysis

**Top Diseases and Biological Functions**		
**Diseases and Disorders**		
**Name**	**P value**	**Number of molecules**
Neurological Disease	2.37E-04 - 7.10E-09	500
Cancer	2.58E-04 - 2.53E-08	2162
Gastrointestinal Disease	1.93E-04 - 2.53E-08	1865
Hepatic System Disease	7.09E-05 - 2.53E-08	998
Organismal Injury and Abnormalities	2.58E-04 - 2.53E-08	2198
**Molecular and Cellular Functions**		
**Name**	**P value**	**Number of molecules**
Cell Morphology	2.37E-04 - 4.21E-14	597
Cellular Growth and Proliferation	2.43E-04 - 2.65E-11	648
Cellular Development	2.43E-04 - 2.05E-10	707
Cellular Movement	2.37E-04 - 1.36E-09	514
Cellular Assembly and Organization	2.52E-04 - 5.50E-09	377
